# Selected genes of Human herpesvirus-8 associated Kaposi’s sarcoma among patients with Human Immunodeficiency Virus-1 and Acquired Immunodeficiency Disease Syndrome

**DOI:** 10.11604/pamj.2019.32.215.17322

**Published:** 2019-04-30

**Authors:** Rodgers Norman Demba, Nathan Shaviya, Sylviah Mweyeli Aradi, Walter Mwanda

**Affiliations:** 1 School of Health Sciences, Kisii University, Kisii, Kenya; 2 Institute of Tropical and Infectious Diseases, University of Nairobi, Nairobi, Kenya; 3 Masinde Muliro University of Science and Technology, Kakamega, Kenya; 4 Department of Internal Medicine, University of Nairobi, Nairobi, Kenya

**Keywords:** KSHV, histology, nested PCR

## Abstract

**Introduction:**

Kaposi's sarcoma (KS) is a kind of cancer that causes flat or raised lesions containing Human herpes virus 8 (HHV8). The KS lesions are common among immunosuppressed HIV patients. Highly Active Antiretroviral (HHART) treats and prevents the development of KS. The objective of this study was to determine the presence of K1 and K15 (predominant alleles) genes in Kaposi's sarcoma-associated herpes virus (KSHV) among immunosuppressed patients due to HIV-1.

**Methods:**

This was a cross-sectional descriptive study where consecutive sampling technique was adopted to pick archived tissue blocks from the Thematic Unit of Anatomic Pathology, Department of Human Pathology, College of Health Sciences, University of Nairobi and Department of Laboratory Medicine, Histology Section, Kenyatta National Hospital.

**Results:**

Upon staining 81 tissue blocks with H & E, 84% (68/81) were diagnosed as KS and 16% (13/81) as KS-like. The K1 and K15 (P) genes were both detected at 88.9% (72/81) in the tissue blocks, with 95.8% (69/72) detection from KS and 4.2% (3/72) from the KS-like.

**Conclusion:**

The K1 and K15 (P) genes of KSHV were present among the immunosuppressed patients with Human Immunodeficiency Virus (HIV)-1. It is important to carry out K1 and K15 (P) genes detection on tissues that are diagnosed as KS or KS-like by histology technique.

## Introduction

Current studies indicate that all types of Kaposi's sarcoma (KS) are caused by Human Herpes Virus (HHV)-8 infections, and the difference in them is attributed by the contributions of many cofactors [1]. Genetic or environmental factors are essential for progression of KS resulting to heterogeneous circulation of the virus in different populations [2]. Immunosuppression due to Human Immunodeficiency Virus (HIV)-1 can result in the manifestation of Kaposi's sarcoma tumors [3]. The genome of HHV-8 is about 140.5 Kilo base pair, and encompasses several terminal repeats [4]. The naming of HHV-8 genes is based on the genomic location, and the letter 'K' is deduced from Kaposi's sarcoma human virus (KSHV) [5]. The HHV-8 genes have been observed to play a role in the KS pathogenesis. K1 gene stimulates and augments other cells in a paracrine manner. Manifestation of KS is heightened when K1 gene which encodes for inflammation unites with the HIV-1 Tat protein [6]. K15 gene is located at the right end of HHV-8 genome and occurs in two alleles as P (Predominant) and M (minor). It has been shown to contribute to the angiogenic properties of the KSHV [7]. Viral pathogenesis of HHV-8 associated KS has been attributed to the interactions between host immune responses, K1 and K15 genes. In addition, K1 and K15 genes have also been detected in cells during latency period and have been linked to viral neoplasia [8]. The geographical distribution of HHV-8 has been well defined. However, what remains a puzzle is the reason for its high prevalence in Africa and Middle East and a relatively lower prevalence in United States and Northern Europe [9]. Studies have suggested the possibility of HHV-8 being transmitted through oral-fecal route but this still yet to be established [10]. The precise mode of transmission of HHV-8 is still not known but, the virus has been successfully detected in semen and saliva [11]. It is not recommended to test children and adults on a routine basis, for this reason the serological status of HHV-8 in patients infected with HIV is frequently unknown [12]. Primary diagnosis of KS is achieved by describing the morphological characteristics of the tumor using Haematoxylin and Eosin (H & E) staining technique [13]. Histological examination has shown that KS is a complex lesion and the presentation is made up of patch stage, plaque stage and a nodular stage [14]. Molecular technique is the recommended diagnostic method for confirmation of KS [15] and it is on this basis that this study was designed to detect K1 and K15 (P) genes of Human herpes virus-8 associated Kaposi's sarcoma among HIV-1 and Acquired Immunodeficiency Disease Syndrome (AIDS) patients using nested polymerase chain reaction (PCR) technique.

**Hypothesis:** the existence KSHV gene K1 and K15 (P) gene is associated with KS among patients who had HIV-1 and AIDS.

## Methods

This was a cross-sectional descriptive study where 104 formalin-fixed paraffin-embedded (FFPE) tissues blocks were retrieved from the Thematic Unit of Anatomic Pathology, Department of Human Pathology, College of Health Sciences, University of Nairobi and Department of Laboratory Medicine, Histology Section, Kenyatta National Hospital between the year 2013 and 2016. The demographics, HIV-1 status, CD_4_ cell count, tumor location, number of tumors, and antiretroviral treatment status of the patients whose tissue blocks had been collected and used were obtained from the registry records in the cytology and human pathology department. For this study, the inclusion criteria were tissue blocks that were previously diagnosed as KS or KS-like with a comprehensive bio-data information linking the blocks and registry records. Tissue blocks previously diagnosed as KS or KS-like that had missing or incomplete bio-data information from the registry records were excluded from the study. Consecutive sampling technique was used to pick a total of 81 tissue blocks from the 104 FFPE because they had met the inclusion criteria. A rotary microtome was used to section FFPE blocks.

**Tissue sectioning:** different blades per FFPE block were used to prevent carry-over of DNA. After each section, the surface of the microtome was sterilized using DNAZap^TM^ PCR DNA (Thermo Fisher Scientific Company Cat No./ID: AM9890) degradation solutions. One tissue section was cut up to 10 µm thick. The tissue sections were processed for Hematoxylin and Eosin (H and E) staining and the results reported by a qualified human pathologist.

**DNA extraction:** DNA extraction was carried out from the sectioned tissues using Qiagen kit GeneRead DNA FFPE (Qiagen^®^company Cat No./ID: 180134).The extraction kit removes paraffin and reverses formalin cross-links from the DNA tissues before it is bound to the QIAampMinElute column. The eluted DNA was then ready to be used for nested PCR.

**DNA detection:** taq PCR Core Kit-Qiagen (Qiagen^®^ company Cat No./ID: 201223) was used to detect K1 and K5 (P) gene. The two (K1 & K15 (P)) selected genes of KSHV were detected using two set of primers. The set of primers used were; K1 product size 868 bpK1a-f ATGTTCCTGTATGTTGTCTGC; K1a-r AGTACCAATCCACTGGTTGCG K1 product size 840 bp K1b-f GTCTGCAGTCTGGCGGTTTGC; K1b-r CTGGTTGCGTATAGTCTTCCG, K15 (P) product size 365 bp K15P-OF TGCAGGCTTGGTCATGGGTTAC; K15P-OR GGGACCACGCTGCAATTAAATG; K15 (P) product size 285 bpK15-3C ACGCATACATGTACTGCCAC; K15-4C CTTTGATATTGCCAGTGGTG. The PCR cycling condition of all the two targeted KS regions were similar and it consisted of 30 number of cycles which entailed; initial denaturation at 94°C for 3 minutes, denaturation at 94°C for 1 minute, annealing at 63°C for 1 minute, extension at 72°C for 1 minute and final extension at 72°C for 10 minutes. The amplified PCR products were analyzed by electrophoresis on a 1% agarose gel containing ethidiumbromide (1µl/ml of agarose solution) and were visualized under ultraviolet light alongside 1Kb DNA ladder. A known case of KS was used as positive control and RNase free water used as a negative control.

**Ethical clearance:** authors of this manuscript obtained ethical clearance for the study from Kenyatta National Hospital and University of Nairobi (ethical clearance number: P682/11/2014).

**Statistical analysis:** sample size of 81 tissue blocks was calculated using the standard statistical formula [16]

$$n=\frac{z^{2}pq}{d^{2}}$$

where Z^2 = 1.96, p = 5.6% based on prevalence HIV infection, trends, and risk factors among persons aged 15-64 years in Kenya: results from a nationally representative study [17], q = 0.944, d^2 = 0.05^2, n = 1.962 X ((0.056) X (1-0.056))/(0.05)^2 = 81. The data was analyzed using SPSS version 21 where the relationship between K1, K15 (P) genes and clinicopathologic factors were tested by chi-square, multiple logistic analysis and *t-test*. A P value < 0.05 was considered statistically significant.

## Results

**Figure 1:**
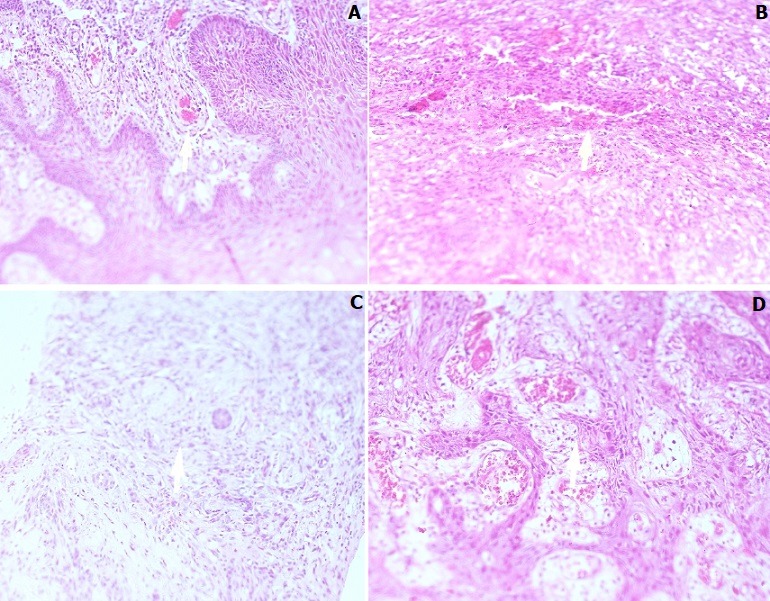
Microscopic identification of Kaposi's sarcoma (KS) and KS-like using H and E staining, X 20; (A) patch: patch stage Kaposi sarcoma showing vessels protruding into a larger vascular space characteristic of the promontory sign; (B) nodular: fascicles of relatively monomorphic spindled cells, with slit-like vascular channels containing erythrocytes; (C) plaque: plaque stage KS with bizarre vessels; there is erythrocyte extravasation and hemosiderin pigmentation; (D) KS-like: morphological feature suggestive of KS

The K1 and K15 (P) genes detected as positive were 88.9% (72/81) and negative was 11.1% (9/81). Among the 88.9% tissue bocks that were positive for the targeted HHV-8 genes, 41.7% (30/72) were from female and 58.3% (30/72) male. All the subjects whose tissue blocks were retrieved for this study were found to be HIV positive. The antiretroviral status of the subjects was also determined, and it showed that out of the 88.9% that had K1 and K15 (P) genes detected, 95.8% (69/72) were on antiretroviral treatment and 4.2 (3/72) were HAART naïve. The CD_4_ counts of the subjects whose tissue blocks were retrieved from the records demonstrated that none of the recruited subjects had a CD_4_ count of above 350 cell/ mm^3^. The number of CD_4_ count was grouped into two groups, 250 being the cut-off point. Among the tissue blocks that had the K1 and K15 (P) genes detected 51.4% (37/72) had CD4 count above 250 cells/ mm^3^ and 48.6% (35/72) had CD4 count below 250 cells/ mm^3^. The distribution of age and CD_4_ cell count were significantly skewed and were bimodal among those who had the K1 and K15 (P) genes detected at a P value of 0.039 and 0.004 respectively (Shapiro test of normality). The records also indicated the distribution and the number of KS lesions among these subjects whose tissue samples were archived as FFPE blocks. The number of KS lesions in these subjects was grouped into two, above 10 and below 10. The tissue blocks that the K1 and K15 (P) genes were detected and had > 10 KS lesions was 55.6% (40/72) and < 10 KS lesion 44.4% (32/72). The distribution of KS was grouped as either localized or generalized, with 37.5% (27/72) localized and 62.5 (45/72) generalized among those that the K1 and K15 (P) genes were detected. The distribution of the KS lesions among the tissue blocks that had the two targeted genes detected were as follows: skin 31.6% (23/72), lower limbs 27.8% (20/72), lymphedema 16.7% (12/72), upper limbs 13.9% (10/72), palate 5.6% (4/72), trunk 1.4% (1/72), genitals 1.4% (1/72) and eye 1.4% (1/72). The study did histology screening on the retrieved tissue blocks to describe the morphology of the KS lesions. Tissue blocks were processed, sectioned, stained by H & E, results read by qualified pathologist and were described as patches, plaques and/or nodules (Figure 1). In this study, among the tissue blocks that had K1 and K15 (P) gene detected, histology results showed that 22.2% (16/72) were plaques, 8.3% (6/72) patches and 69.4% (50/72) nodules. The tissue blocks that were diagnosed as KS-like were 16% (13/81) out of which 4.2% (3/72) had the two target genes detected. Multiple logistic regression analysis (Table 1) was done to determine the prevalence odds ratio and this model was found to be significant at P value of < 0.007.

**Table 1:** Estimated of crude prevalence odds ratio (POR) using simple logistic regression for K1 and K15 (P) genes

Characteristics	n (%)	K1 & K15 (P) gene positive n (%)	POR	95% CI	P value
**Gender**			0.57	0.14 - 2.31	0.428
Male	46(56.8)	42(58.3)			
Female	35(43.2)	30(41.7)			
**Age**			1.07	0.99 – 1.18	0.104
18-29yrs	9(11.1)	7(9.7)			
30-39yrs	39(48.1)	34(47.2)			
40-49yrs	23(28.4)	21(29.2)			
50-59yrs	6(7.4)	6(8.3			
60 yrs & above	4(4.9)	4(5.6)			
**On treatment**			0.35	0.03- 3.75	0.377
On ARVs	77(95.1)	69(95.8)			
HAART naive	4(4.9)	3(4.2)			
**Number of lesion**			1.0	0.25- 4.03	1.014
>10	44(54.3)	39(54.2)			
**<**10	37(45.7)	33(45.8)			
**Distribution of lesion**			0.21	0.03- 1.76	0.152
Generalized	53(65.4)	45(62.5)			
Localized	28(34.6)	27(37.5)			

*Antiretrovirals (ARVs), Highly Active Antiretroviral Therapy (HAART), % (percentage), > above and < below

## Discussion

Genotyping analysis in this study revealed that the Kaposi's sarcoma-associated herpes virus (KSHV) genes, K1 and K15 (P) were detected among the selected tissue blocks. These findings affirm the earlier hypothesis that K1 and K15 (P) genes of Kaposi's sarcoma-associated herpes virus (KSHV) were present in the tissue blocks retrieved from patients with HIV-1 and AIDS. A previous study [18] conducted at the same facility looked at the prevalence of KS and it was noted that the existence was ranging from 2-5%. The discrepancy could be attributed to the number of cases as the previous study had total of 1108 cases of KS consisting of 911 males and 197 females. It is worth noting that unlike the former study that used clinical and histological diagnosis, the current study adopted the use of a more sensitive test that detected HHV-8 Deoxyribonucleic acid (DNA). In Morocco, an epidemiology study on Kaposi's sarcoma showed that they successfully detected the K1 gene at 870 bp in 35/35 DNA samples and K15 (P) was spotted in 31/35 using 362 bp PCR product [19]. In Zambia, the K1 sequences were detected in blood from HIV positive febrile infants who had KS. The study went further and did a distinct grouping of KS strains and it was noted that K1 glycoprotein was highly variable [20]. A study on the evolution of K15 gene and HHV-8 recombination was done in Uganda and the findings indicated that K15 was highly divergent with P allele being predominant in 30/30 KS patients. The whole K15 gene (2101 bp) was used in the analysis to establish the existence of HHV-8 [21]. In Zimbabwe, the existence of K1 diversity was evaluated and it was observed that there was no significant relationship that was found to exist amongst K1 subtypes and the clinico-demographic features among the 65 AIDS-KS patients [22]. The results of this study will add to the literature on the existing list of countries that have successfully detected K1 and K15 (P) genes associated with AIDS-KS in Africa. The crude prevalence odds ratio (POR) from Table 1 indicated that none of the variables were found significant (P < 0.05); but some variables like gender, CD_4_ cell count, treatment and distribution of KS lesions showed a strength in the association: gender for example, we noticed that compared to females, males were twice represented among those with K1 and K5 (P) genes cases.

These findings were in agreement with another study [23] where it was observed that in Africa, tumors associated with KS were likely to be diagnosed in male patients. From the registry records where the bio-data and clinical information of the patients, whose tissue blocks were used in this study, none had a CD4 count above 350 cell/ mm^3^. Other studies [24, 25] have also indicated that CD4 cell count <350 cells/ mm^3^ are linked to high risk of developing KS whether one is on HIV treatment or not. However, recommendations have been made on the need to lookout on KS cases in subjects with high CD4 cell count [24]. For treatment, HAART naive group in this study were found to be twice represented among the K1 and K5 (P) genes cases compared to those on treatment. Typically, when a patient with AIDS associated KS adheres to antiretroviral therapy (ART), their CD_4_ cell count is elevated and clinical presentation of KS tumors either stabilize or resolve [26]. However, when a patient is on ganciclovir (an antiviral used in the treatment of various forms of herpes) the replication of KSHV is inhibited [27], although after the manifestation of KS tumors these drugs may not be of use. Confirmed AIDS-KS patients should be on highly active antiretroviral therapy (HAART) to achieve 60-90% remission [28]. The most likely reason why subjects in this study had KS presentations despite being on ART could be non-adherence or antiretroviral resistance. Kaposi's sarcoma tumors resulting from HIV and AIDS are known to be severe, resistant and are caused by HHV-8 [29]. Typical KS lesions include macules, papules and nodules. However other morphological variants that have been used to describe it are: patches, plaques, exophytic, infiltrative, keloidal, telangiectatic, cavernous, ulcerative, bullous and verrucous types [30]. For this study, the histological morphology of the KS lesions was nodular, plaques and patches. Majority of the morphological cases were nodular. The distribution of KS in terms of histological morphology was consistent with similar studies that were conducted in Tanzania [31] and Zambia [32] where it was observed that nodular had the most distribution at 68.7%, (82/120) and 60.7% (51/84) respectively. In this study, among those who presented with nodular morphology of KS 61.2% (30) were males and 38.8% (19) females. These findings were consistent with a study conducted in Uganda [33] where women were less likely to have nodular lesions of KS compared to men. In the Uganda study, reduced rate of KS among females might have been due to gender related factors that include hormonal, environmental or genetic factors.

## Conclusion

In conclusion, Kaposi's sarcoma-associated herpes virus (KSHV) genes, K1 and K15 (P) were detected among tissue blocks collected from patients infected with HIV-1 and AIDs. The morphology of KS lesions upon histological diagnosis was distributed as patches, plaques and nodules. East Africa is traditionally known to have a high prevalence of KS [34]. The precision of pathological diagnosis of KSHV should incorporate skin punch biopsy and HHV-8 LANA-1 (Latency- associated nuclear antigen-1).

### What is known about this topic

- In resources limited health facilities, Kaposi's sarcoma is routinely diagnosed by clinical presentations and cytological identification using H & E staining technique.

### What this study adds

- There is need to detect HHV-8 using a robust diagnostic techniques to aid a precise patient management because the patients in this study had a dark skin pigmentation and in another study conducted on dark-skinned patients, KS had been confirmed to mimic a number of non-KS like dermatological conditions;
- It further stated that the patients who presented with violaceous skin lesions were likely to be considered a suspected case of KS which is difficult to demonstrate in dark-skinned individuals.

## Competing interests

The authors declare no competing interests.
